# An Oakum Respirator

**Published:** 1871-11

**Authors:** Somerville Oliver


### Miscellaneous.

### An Oakum Respirator.

By Somerville Oliver, M.D.

Having witnessed the excellent results of the application of carbolic acid and also of oakum in the treatment of wounds and other lesions, it occurred to me that, in cases of phthisis and chronic bronchitis attended with purulent or muco-purulent expectoration, and also in cases of pyæmia, the purulent or muco-purulent matter would probably be diminished, and the condition of the patient otherwise improved, by the habitual use, for a time, of a "respirator," (if I may so term it) consisting of fresh oakum, or woollen cloth impregnated with carbolic-acid solution. The idea was still more impressed on my mind when a non-professional friend in Glasgow incidentally told me that those persons engaged in splitting rosin-barrels into fragments for firewood are remarkably exempt from disease, especially of the zymotic class; and that they themselves attribute their comparative freedom from disease to their special vocation.

I therefore made a rude sort of "respirator" by filling a small net (such as is worn by ladies over their chignons) with oakum; and in a few cases in which I have experimented with it, benefit has, I think, obviously accrued. Perhaps some of my professional brethren who have better opportunities than myself may make trial of the oakum or carbolic respirator, and observe the result. It may even prove useful in empyema, when the pus has found its way from the pleural cavity into the lung, in the usual way. I tried oakum rather than carbolic acid simply because I believe that the creasote of the tar acts at least as energetically as carbolic acid in destroying the minute putrefactive organisms of the atmosphere during their passage through the respirator towards the lungs, and because the oakum does not require to be changed so frequently as the woollen cloth or lint impregnated with carbolic-acid lotion.

My reason for suggesting pyæmia as a case in which a carbolic acid or oakum "respirator" may be used is, that I imagine that the inspiration of either of the antiseptics may in some measure prevent further deterioration of the blood, and also of the purulent deposits in various parts of the body, especially in the lungs. Instead of wearing a respirator at night, a quantity of oakum in a net may be merely placed on the pillow before the patient. And, though oakum or carbolic acid be used as advised, other means need not be set aside in the treatment of any case.

Oakum and carbolic acid appear to be useful chiefly in those conditions where there is a tendency to production of pus or actual formation of it; and this seems to be the reason why oakum and carbolic-acid solution are apparently less valuable when applied to the indolent ulcer than they are to most other sores. The *pressure* of the *callous margin* of the indolent ulcer interferes with the production of healthy granulations, or prevents their formation altogether; consequently it also interferes with the production, or entirely prevents the formation of pus; and the way in which the pressure of the callous margin of the ulcer prevents the formation of healthy granulations is indicated by the condition of the eye known as chemosis. In chemosis the cornea and its conjunctival layer are impaired or destroyed, it may be, chiefly by the obstruction of the circulation in the bloodvessels at the *margin* of the cornea; and in the case of the indolent ulcer, granulations are either not developed, or they are usually pale and flabby, owing in a great degree to the pressure of the callous skin around the sore on the subjacent *superficial capillaries* interrupting the flow of blood through them, and so interfering with the due nutrition of the ulcerated *surface.—Lancet.*

